# The spatial segregation patterns of sharks from Western Australia

**DOI:** 10.1098/rsos.160306

**Published:** 2016-08-17

**Authors:** Matias Braccini, Stephen Taylor

**Affiliations:** Western Australian Fisheries and Marine Research Laboratories, PO Box 20, North Beach, Western Australia 6920, Australia

**Keywords:** conservation, fisheries management, sustainability

## Abstract

The extent to which sharks segregate by size and sex determines the population structure and the scale at which populations should be managed. We summarized 20 years of fisheries-dependent and independent sampling to define the spatial patterns of size and sexual segregation for sharks in Western Australia. *Carcharhinus obscurus* and *C. plumbeus* showed a large-scale (more than 1000 km) latitudinal gradient in size. Large individuals occurred predominantly in the northwest and north whereas smaller individuals occurred predominantly in the southwest and south. *Mustelus antarcticus* and *Furgaleus macki* showed strong sexual segregation at very large scales. Females occurred predominantly in the west and southwest whereas the proportion of males in catches substantially increased in the southeast. The populations of other shark species did not show sex and size segregation patterns at very large scales; most species, however, showed varying degrees of segregation when data were analysed at a smaller scale. These findings highlight the importance of matching the scale of observation to the scale of the phenomenon observed. As many shark species are highly mobile, if sampling is opportunistic and constrained both temporally and spatially, the observed segregation patterns may not be representative of those at the population level, leading to inaccurate scientific advice.

## 1. Introduction

The movement patterns of sharks are varied, ranging from hundreds of metres (e.g. [1]) to thousands of kilometres (e.g. [2]). These movements shape the population structure of sharks and the extent to which species are exposed to fishing. Understanding population structure therefore assists in defining boundaries for spatially structured models and fisheries management.

In Western Australia (WA), commercial fisheries that target sharks have been regulated through a range of fishing effort controls, gear restrictions and fishing closures [3]. Although in other parts of the world unregulated and unrestricted fishing has led to drastic declines in shark populations [4], the population status of the main commercial shark species in WA is either adequate or recovering [3]. Further improvement to the management of sharks in WA and elsewhere, however, needs to consider patterns in population structuring. For example, the same unit of fishing effort can have different effects on a population if exerted in an area with a high concentration of large mature females as opposed to an area with a more even distribution of sizes and sexes (e.g. [5]).

Size and sex segregation is a widespread behaviour among sharks with many species showing different degrees of segregation (e.g. [6]). The scale of sampling, however, can influence the segregation pattern detected. For example, sampling over a short period of time may reveal a spatial pattern of segregation that is temporary and not representative of the population over a longer time frame [7]. In addition, if the scale of observation is considerably smaller than the scale of the population structure, this could also lead to inaccurate information on the population structure being used in population assessments. This study therefore summarizes 20 years of sampling effort across a very large spatial scale to assist in understanding the spatial patterns in size and sex ratio for sharks of the west coast of Australia.

## 2. Material and methods

**Figure 1.**
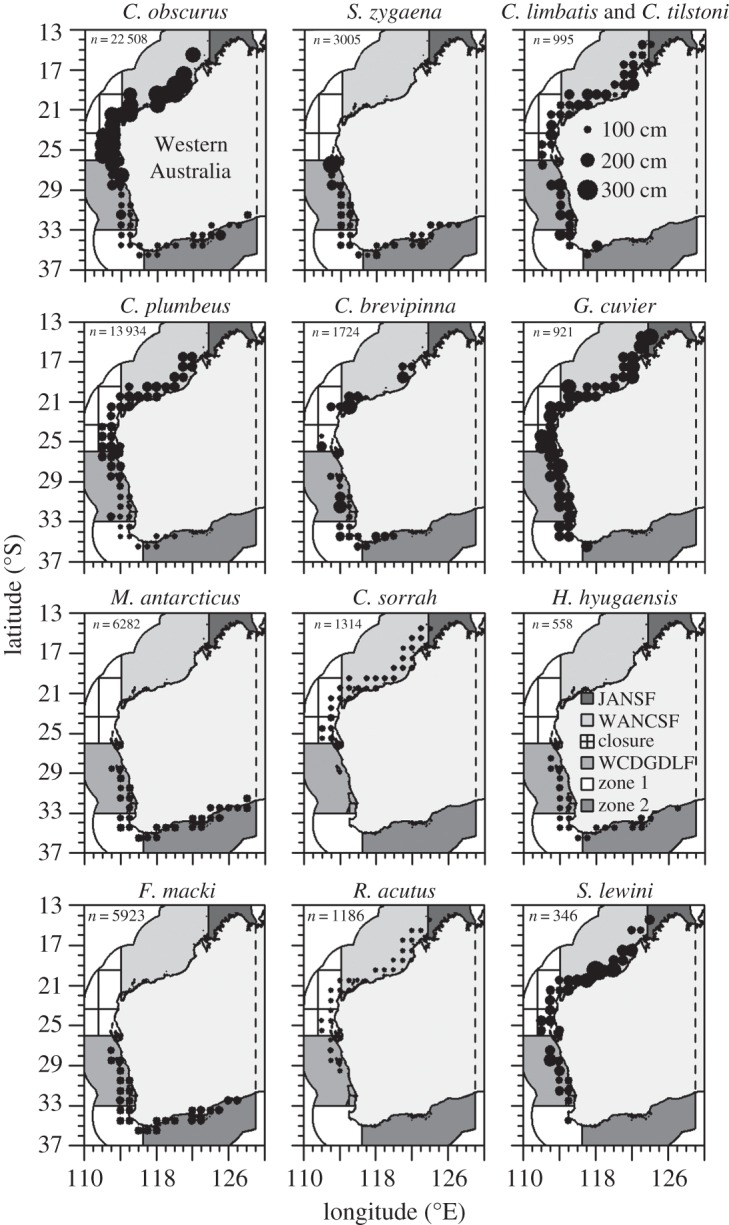
Spatial patterns in the observed fork length (FL) by management zone. Observations were grouped into spatial blocks of 1° latitude by 1° longitude. Dot size represents the mean size per block.

Sharks were sampled by scientifically trained observers between 1993 and 2013 on-board commercial fishing and research vessels using gillnets (6 and 7 inch mesh size) and longlines across WA (figure 1). Gear was deployed during day and night in depths of between 1 and 230 m for time periods of between 1 and 26 h. For each gear deployment, the date, time, GPS location and bottom depth (in metres) were recorded. Upon gear retrieval, all individuals were sexed and identified to the species level, and their fork length (FL) was measured (in centimetres). Separation of the closely related *C. limbatus* and *C. tilstoni* in the field was impractical, as the most useful diagnostic feature (counts of precaudal vertebrae) could not be taken. The two species are thus referred to as ‘*C. limbatus* and *C. tilstoni*’ throughout this paper.

Analyses focused on large-scale spatial patterns in FL and sex ratios, so observations were grouped into the spatial zones used for managing shark fisheries in WA (JANSF: Joint Authority Northern Shark Fishery; WANCSF: WA North Coast Shark Fishery; closure: Ningaloo Closure; WCDGDLF: West Coast Demersal Gillnet and Demersal Longline (Interim) Managed Fishery; zone 1: zone 1 of the Joint Authority Southern Demersal Gillnet and Demersal Longline Managed Fishery, JASDGDLF; zone 2: zone 2 of the JASDGDLF) (figure 1). An orthogonal sampling design was logistically infeasible. To minimize the influence of confounded temporal and spatial effects, analyses focused on species with at least 10 years of sampling per zone, more than 50 observations per zone and more than 100 observations in total. To investigate large-scale patterns, it was necessary to combine samples obtained using longlines (used predominantly in the north and northwest) and gillnets (used predominantly in the west and southwest). The implications of combining information collected using different sampling gear is discussed below.

Generalized linear models were used to determine spatial patterns in FL and sex ratio (response variables) with zone and depth (model predictors). Depth was classed into 10-m bins. Owing to the high correlation between ‘method’ and ‘zone’, ‘method’ was not used as a model term. No interactions were considered due to a lack of observations for most factor combinations. Preliminary analyses showed no obvious differences among sexes or annual trends in FL so neither sex nor year was included as a model predictor. For the analysis of sex ratios, preliminary analysis also showed no particular annual trends so year was not included as a model predictor. For FL, we used a normal distribution whereas for sex ratio we used a binomial distribution as the response variable was the proportion of males. All analyses were done in the statistical package R [8].

## 3. Results and discussion

A total of 76 346 individuals from more than 50 shark species were collected (electronic supplementary material, table S1). *Carcharhinus obscurus*, *C. plumbeus*, *M. antarcticus* and *F. macki* were the top four most caught species. Further analyses focused on the most commonly caught species with the exception of *Heterodontus portusjacksoni*, *Galeorhinus galeus*, *C. amboinensis* and *Orectolobus hutchinsi*, as these species did not meet the criteria considered for reducing the confounded temporal and spatial effects.

Mean FL showed significant differences across zones and depths but the model explained a large part of the total deviance for only a few species (table 1). For *C. plumbeus* and *C. obscurus*, zone explained more than 30% of the total deviance but for the remaining species, it explained considerably less. *Carcharhinus obscurus* and *C. plumbeus* showed a clear gradient in size segregation over a very large scale. For both species, large individuals predominantly occurred in the northwest and north of WA whereas smaller individuals were more prevalent in the southwest and south (figure 1; electronic supplementary material, figure S1). The preference of temperate latitudes by juveniles and of warmer northern latitudes by adult *C. obscurus* and *C. plumbeus* was also noted in [9] and [10]. This information has already been incorporated in the management of these species with a large (approx. 1.3 million km^2^) spatial closure (herein referred to as ‘closure’, figure 1) established in 1993 for protecting the breeding stocks [11]. The remaining species showed no large-scale spatial patterns in mean FL. *Hypogaleus hyugaensis*, *C. obscurus* and *C. brevipinna* showed depth gradients in mean size, with smaller individuals occurring in deeper water for *H. hyugaensis* and larger individuals occurring in deeper water for *C. obscurus* and *C. brevipinna* (electronic supplementary material, figure S2). However, owing to the considerable variability in size for several depth bins, depth only explained a small percentage of the deviance for *C. obscurus* and *C. brevipinna* (table 1). For the remaining species, there were no strong patterns in mean size across depths (electronic supplementary material, figure S2).

**Table 1.** Summary of the generalized linear model fitted to the fork length data; *p*-values, percentage of deviance explained by each term and the total deviance explained are displayed for each model. Species are ordered by sample size.

	*p*-values		deviance explained (%)		
species	zone	depth	zone	depth	total
*C. obscurus*	<0.001	<0.001	31	5	36
*C. plumbeus*	<0.001	<0.001	56	6	62
*M. antarcticus*	<0.001	<0.001	2	3	5
*F. macki*	<0.001	<0.001	2	2	4
*S. zygaena*	<0.001	<0.001	4	9	13
*C. brevipinna*	<0.001	<0.001	16	10	26
*C. sorrah*	<0.001	<0.001	3	3	6
*R. acutus*	<0.001	<0.001	3	13	16
*C. limbatus* and *C. tilstoni*	<0.001	<0.001	10	19	29
*G. cuvier*	<0.001	<0.001	3	6	9
*H. hyugaensis*	<0.001	<0.001	7	36	43
*S. lewini*	0.013	<0.001	3	27	30

**Figure 2.**
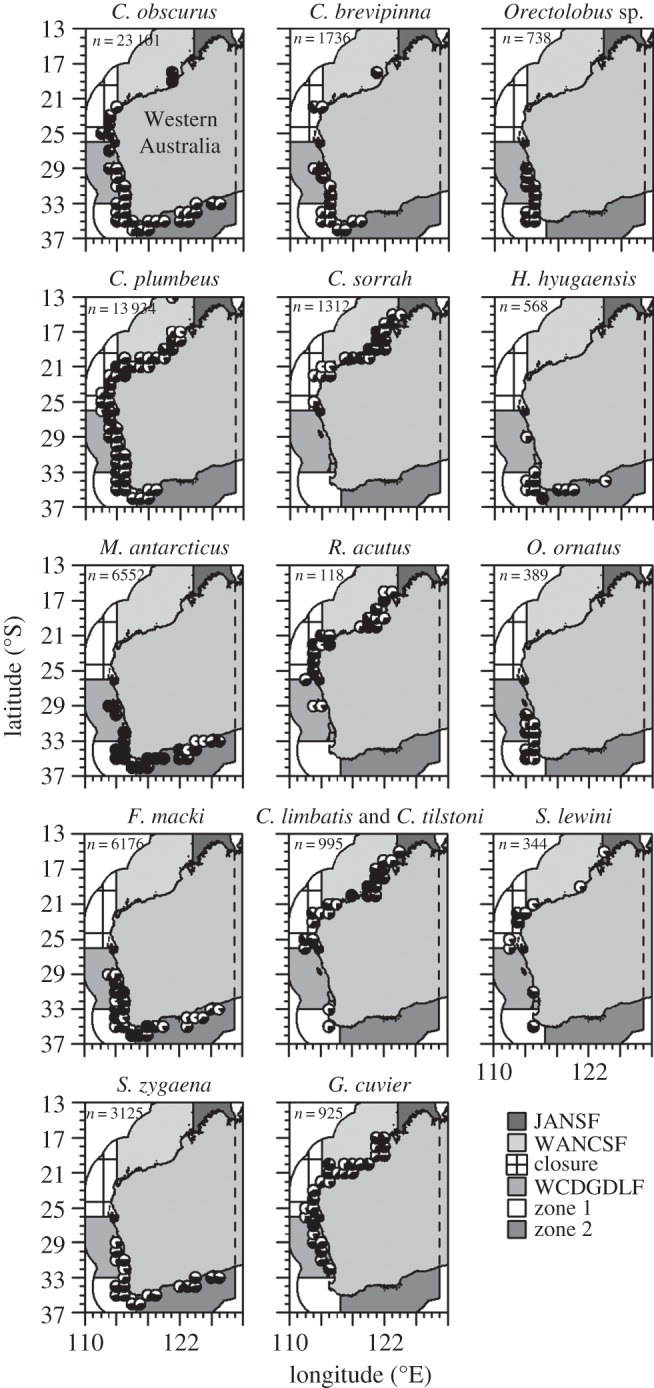
Spatial patterns in the observed sex ratio by management zone. Observations were grouped into spatial blocks of 1° latitude by 1° longitude. The proportion of females and males per block is shown in black and white, respectively, within each circle.

Significant differences in sex ratio were observed across zones and depths and the model explained a large part of the total deviance for most species (table 2). Zone explained a considerable part of the deviance for several species. However, only *M. antarcticus* and *F. macki* showed clear gradients in sexual segregation over a large spatial scale with females occurring mostly in the west and southwest and the occurrence of males increasing towards the southeast (figure 2). As *M. antarcticus* and *F. macki* were mostly sampled by gillnets, which for these species select for large juveniles and adults, the observed sexual segregation patterns relate to these size classes only. No inferences on the sexual segregation of small juveniles could be made from these data. The remaining species showed no large-scale gradients in sex ratio. For *M. antarcticus* and *F. macki*, the proportion of males decreased with depth (electronic supplementary material, figure S3). For *S. zygaena*, *C. sorrah*, *G. cuvier* and *C. limbatus* and *C. tilstoni* the proportion of males increased with depth (electronic supplementary material, figure S3). For the remaining species, there were no clear patterns in the proportion of males across depths.

**Table 2.** Summary of the generalized linear model fitted to the sex ratio data; *p*-values, percentage of deviance explained by each term and the total deviance explained are displayed for each model. Species are ordered by sample size.

	*p*-values		deviance explained (%)		
species	zone	depth	zone	depth	total
*C. obscurus*	<0.001	<0.001	36	41	77
*C. plumbeus*	<0.001	<0.001	14	36	50
*M. antarcticus*	<0.001	<0.001	33	54	87
*F. macki*	<0.001	<0.001	16	50	66
*S. zygaena*	0.011	0.003	18	51	69
*C. brevipinna*	0.246	0.898	16	8	24
*C. sorrah*	<0.001	<0.001	18	47	65
*R. acutus*	<0.001	<0.001	46	28	74
*C. limbatus and− C. tilstoni*	<0.001	<0.001	18	46	64
*G. cuvier*	0.83	0.112	3	47	50
*Orectolobus sp.*	0.188	0.055	15	67	82
*H. hyugaensis*	0.527	0.002	3	65	68
*Orectolobus ornatus*	0.639	0.656	2	54	56
*S. lewini*	0.001	<0.001	27	58	85

Most shark species segregate by size and sex during some point of their life cycle [12]. Spatial segregation can result from a range of reasons such as refuge from mating, intraspecific competition and differences in prey availability and energetic requirements (e.g. [7,13]). Regardless of the drivers for spatial segregation, incorporating this information in the modelling of shark populations can provide a more accurate representation of their population dynamics and the effects of fishing exploitation. For example, sex-biased exploitation may exacerbate population declines [14] as the interaction of complex population structuring with area-specific fishing may have disproportionate effects on different components of a population [7]. Furthermore, incorporating spatially explicit dynamics can improve the way population models fit the available data. For example, a spatially explicit model for school sharks in southeastern Australia that considers the complex spatial structure of this species provided more precise population estimates than the spatially aggregated counterpart [15].

The scale of observation influenced the observed FL and sex ratio patterns. For example, when observed at a smaller scale (one degree of latitude) some of the adjacent latitudes showed different FL distributions for some of the species (figure 2; electronic supplementary material, figure S4) and several adjacent latitudes showed different sex ratios for many species (figure 2). Furthermore, for several species FL (electronic supplementary material, figure S5) and the proportion of males (electronic supplementary material, figure S6) observed per month varied considerably among months. Also, several species showed sexual segregation when analyses were done at the scale of a sampling event. *Carcharhinus plumbeus*, *C. sorrah*, *R. acutus*, *C. limbatus* and *C. tilstoni*, *F. macki* and in particular *M. antarcticus* schooled by sex in 1, 12, 29, 14, 33 and 73% of the sampling events, respectively. However, at a population level (which corresponds to the level of management in general), large-scale patterns in sex ratio were only observed for *F. macki* and *M. antarcticus*.

It was logistically and financially impractical to investigate population-level segregation patterns using a structured survey design over such a large area and time frame. The opportunistic nature of the sampling design thus reflects a compromise between statistical rigour and biological meaning. It was also necessary to combine several years of sampling using demersal gillnets and longlines to maximize spatial coverage and hence to be able to detect large-scale patterns. While the combination of gear types could have introduced bias, the size composition data obtained when gillnets and longlines were used in the same latitudes showed considerable overlap for most species (electronic supplementary material, figure S7). The size composition of survey data results from the size-selectivity effects of the sampling gear used whereas the size composition of the fished population results from the history of size-selective fishing mortality. Hence, there is potential confounding between stock structure and the effects of fishing, particularly for highly selective gear such as gillnets. To some extent, combining multiple years over the exploitation history of the analysed species may reduce this bias. Finally, the models used to study large-scale patterns did not include interactions terms due to the lack of term combinations for most species. This may mask biologically important effects. For example, *G. cuvier* in Hawaii [16] and in this study showed no apparent segregation by depth whereas juvenile *C. plumbeus* in WA [10] and Hawaii [17] occur in deeper waters. In our study, no clear trend between depth and size was observed using the *C. plumbeus* data combined across all years and zones (electronic supplementary material, figure S2); however, data collected only with gillnets in the WCDGDLF zone showed a decreasing trend in size with depth (M Braccini *et al*. 2016, unpublished data). Species-specific analyses could therefore shed light on the segregation patterns of these shark species at a smaller scale.

*Carcharhinus obscurus* and *C. plumbeus* showed large-scale size segregation and *M. antarcticus* and *F. macki* showed large-scale sex segregation. *Carcharhinus obscurus* is capable of long-distance displacements (e.g. [18]) and acoustically tagged individuals frequently undertake round-trip long-distance migrations between northern and southern WA (M Braccini *et al*. 2016, unpublished data). *Carcharhinus plumbeus* has also been documented to be highly mobile; for example, adults annually migrate along the east coast of the US from as far south as the Gulf of Mexico to summer nurseries as far north as off New Jersey [19]. In WA, *C. obscurus* and *C. plumbeus* exhibit considerable spatial segregation. Adults are more abundant north of 26°S latitude and juveniles occur predominantly south of 26°S and slowly migrate northwards to join the breeding stock [20]. The reported movement patterns of *C. obscurus* and *C. plumbeus* are reflected in the clear latitudinal gradient in size observed in this study. In WA, *M. antarcticus* and *F. macki* are less mobile than *C. obscurus* and *C. plumbeus* and males and females show comparable movement patterns (M Braccini *et al.* 2016, unpublished data). Hence, the observed spatial sex segregation would be related to other factors, such as sexual differences in energy requirements associated with growth and reproduction [13], rather than differential movement between sexes.

## 4. Conclusion

Our findings highlight the importance of matching the temporal and spatial scale of sampling to the scale of the phenomenon observed. Sharks are highly mobile and sampling is generally opportunistical. Hence, if a small sampling window is used, segregation patterns at the population scale may be misinterpreted, leading to inaccurate scientific advice.

## Supplementary Material

Figure S1

## Supplementary Material

Figure S2

## Supplementary Material

Figure S3

## Supplementary Material

Figure S4

## Supplementary Material

Figure S5

## Supplementary Material

Figure S6

## Supplementary Material

Figure S7

## Data accessibility

The data accompanying this manuscript have been deposited in Dryad at: http://dx.doi.org/10.5061/dryad.d3t53.
