# Supplementary figures and images for: The spatial segregation patterns of sharks from Western Australia
Source: R Soc Open Sci. 2016 Aug 17;3(8):160306. doi: 10.1098/rsos.160306 (PMC5108959; doi:10.1098/rsos.160306)

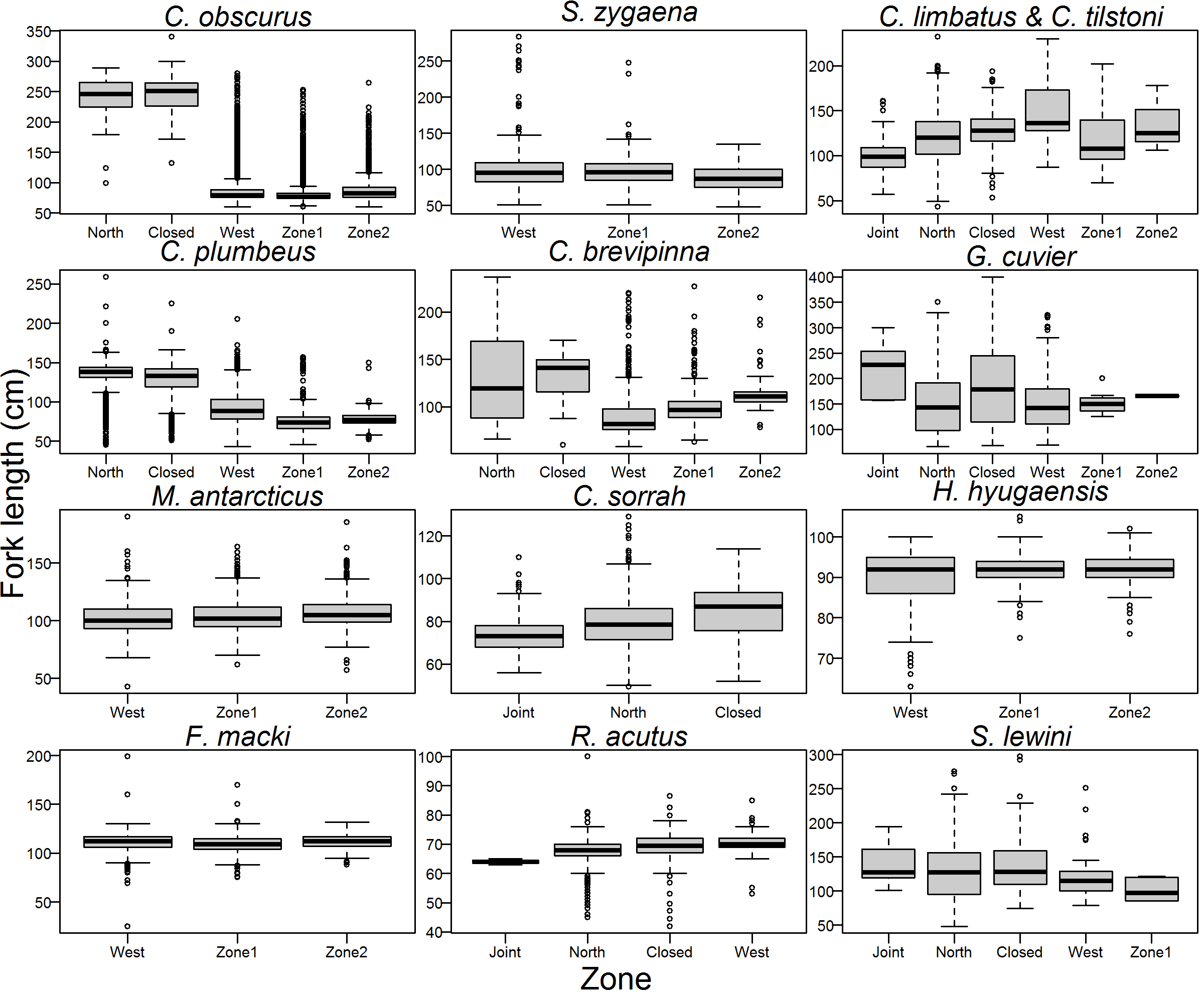

Supplement: Figure S1 [file rsos160306supp1.tiff]

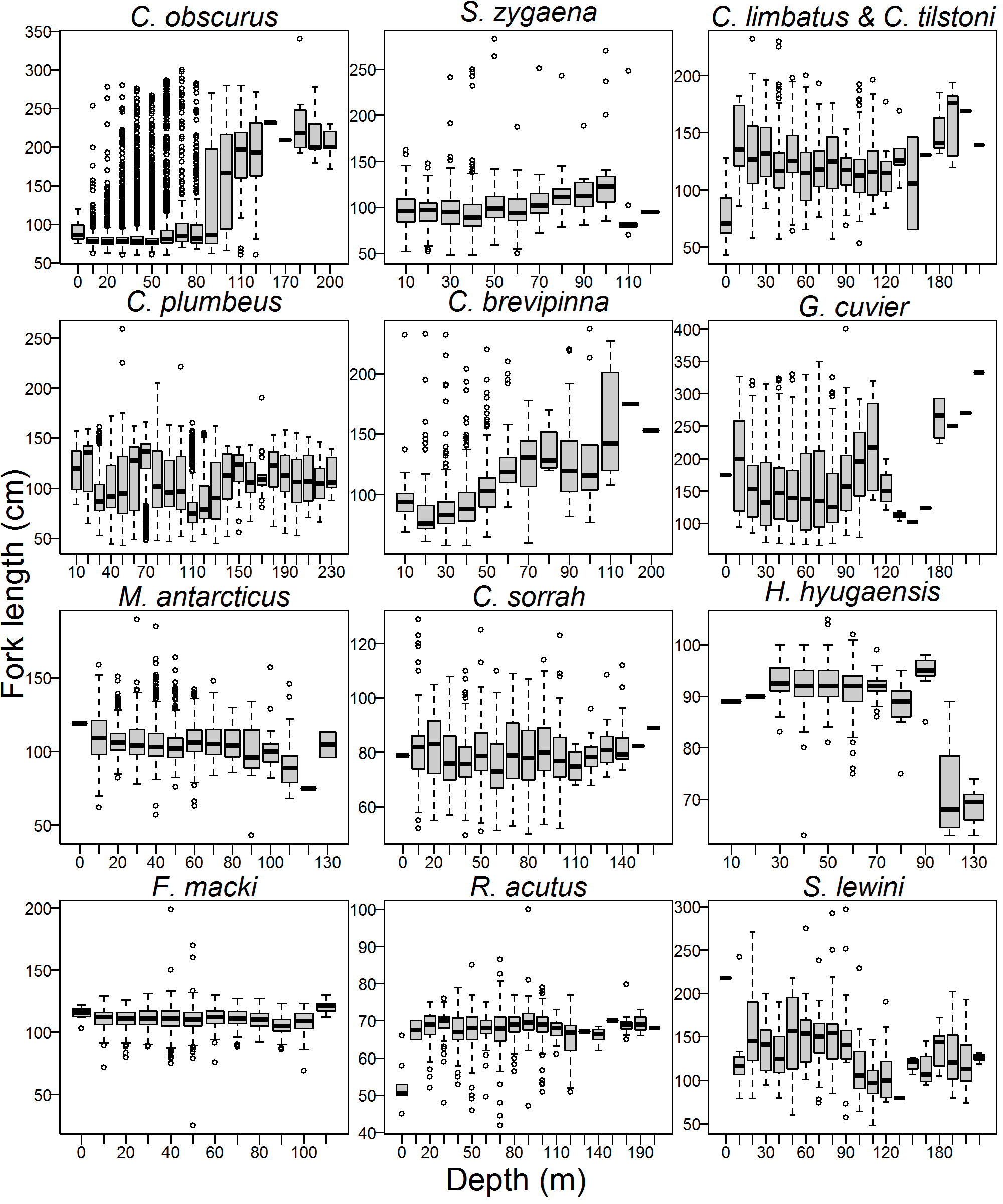

Supplement: Figure S2 [file rsos160306supp2.tiff]

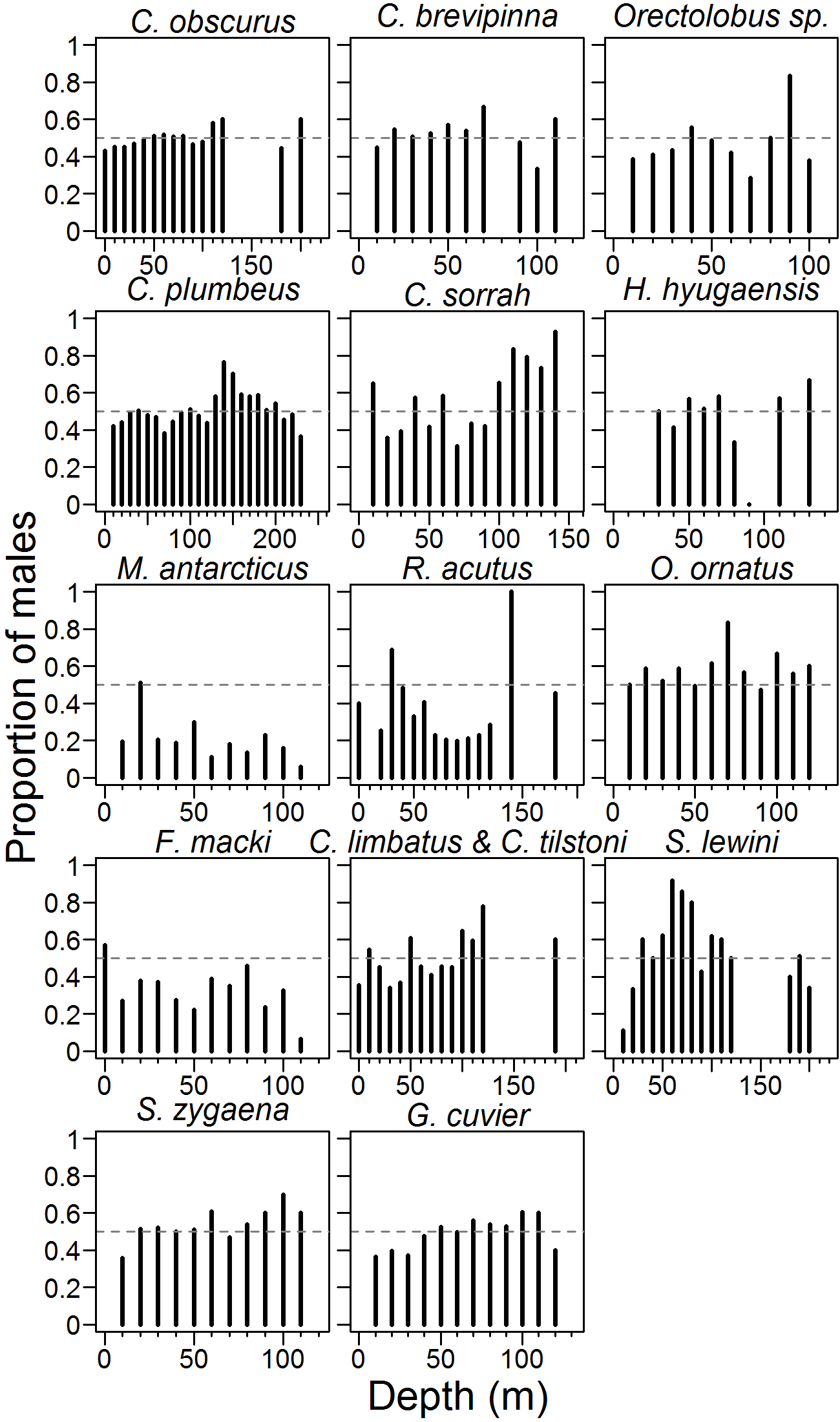

Supplement: Figure S3 [file rsos160306supp3.tiff]

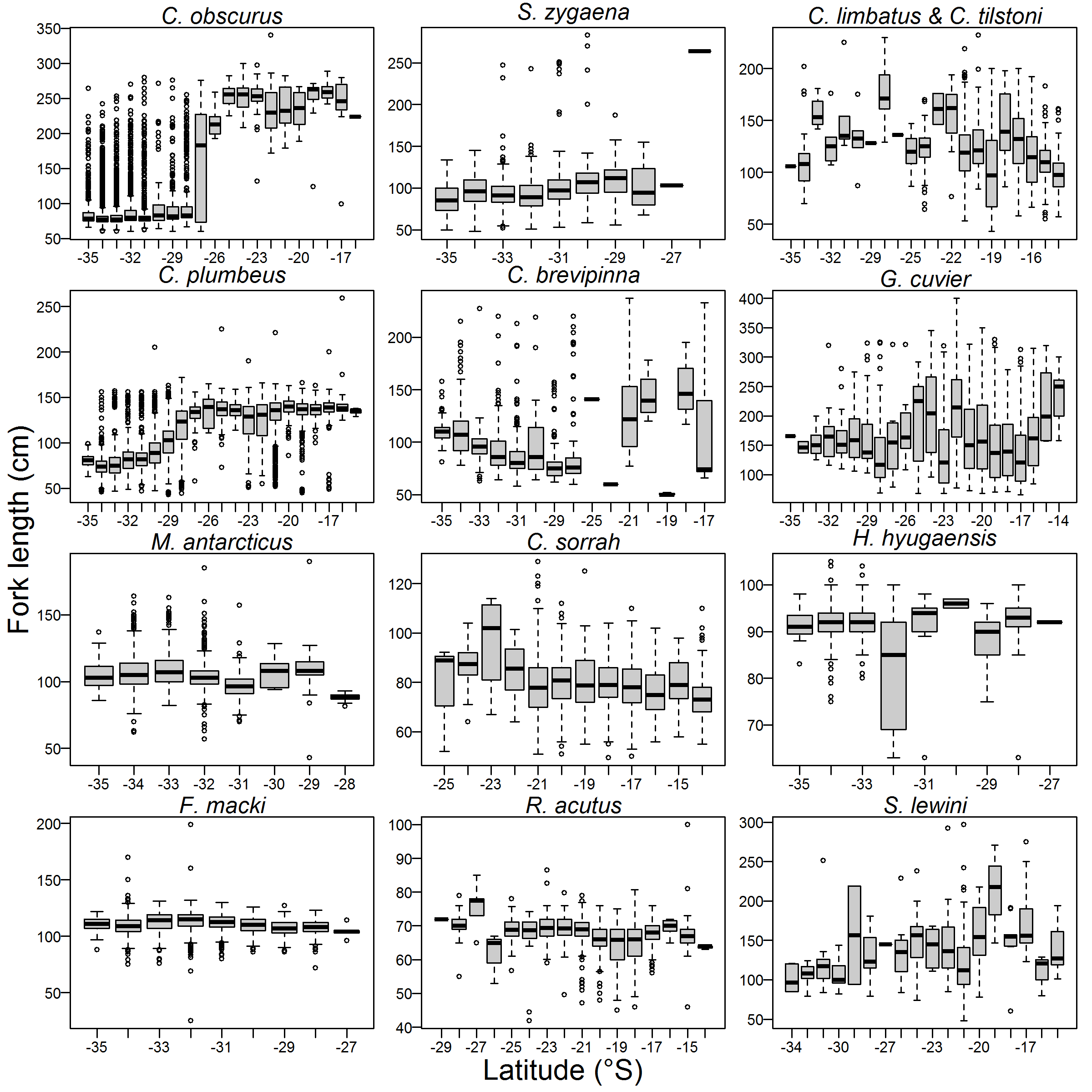

Supplement: Figure S4 [file rsos160306supp4.tiff]

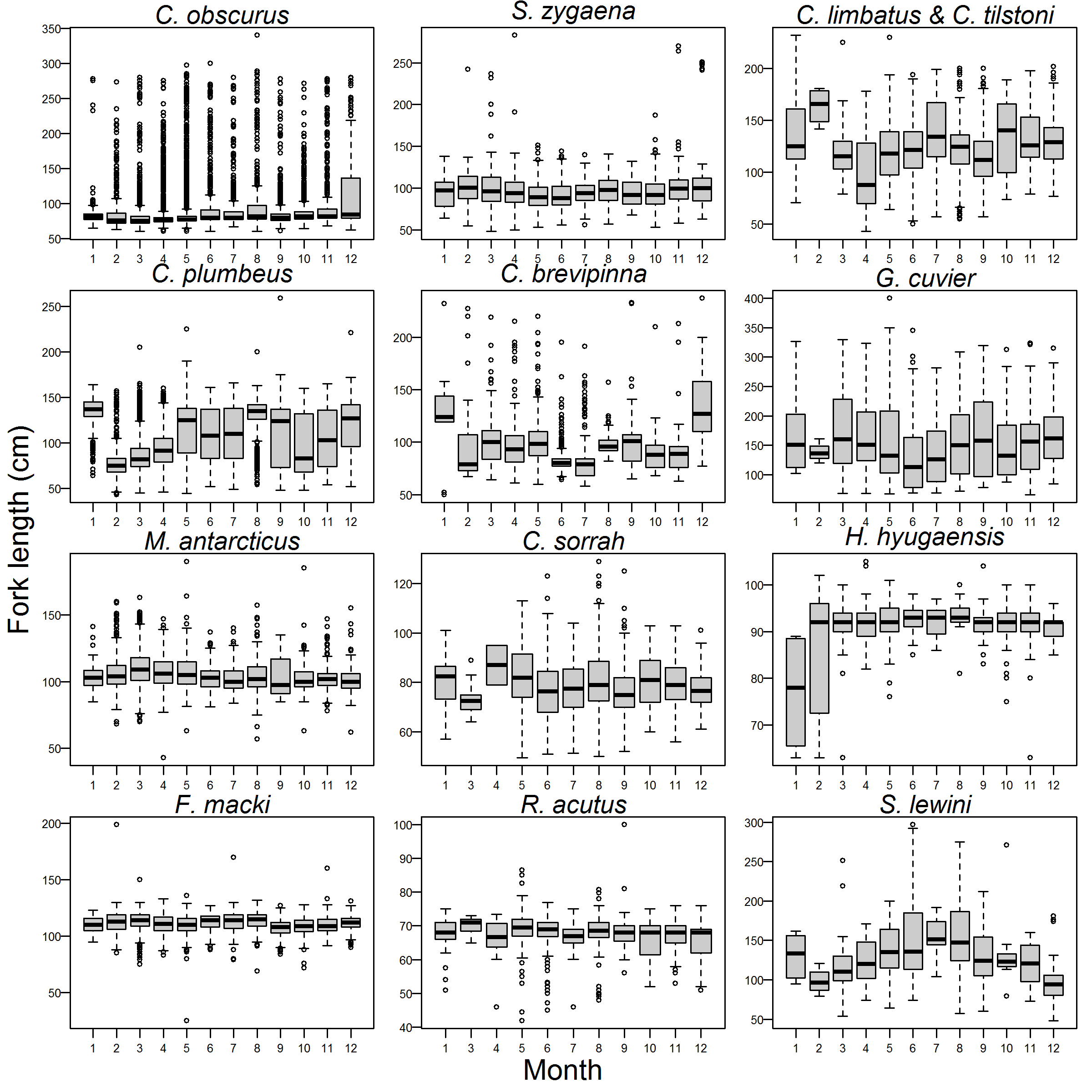

Supplement: Figure S5 [file rsos160306supp5.tiff]

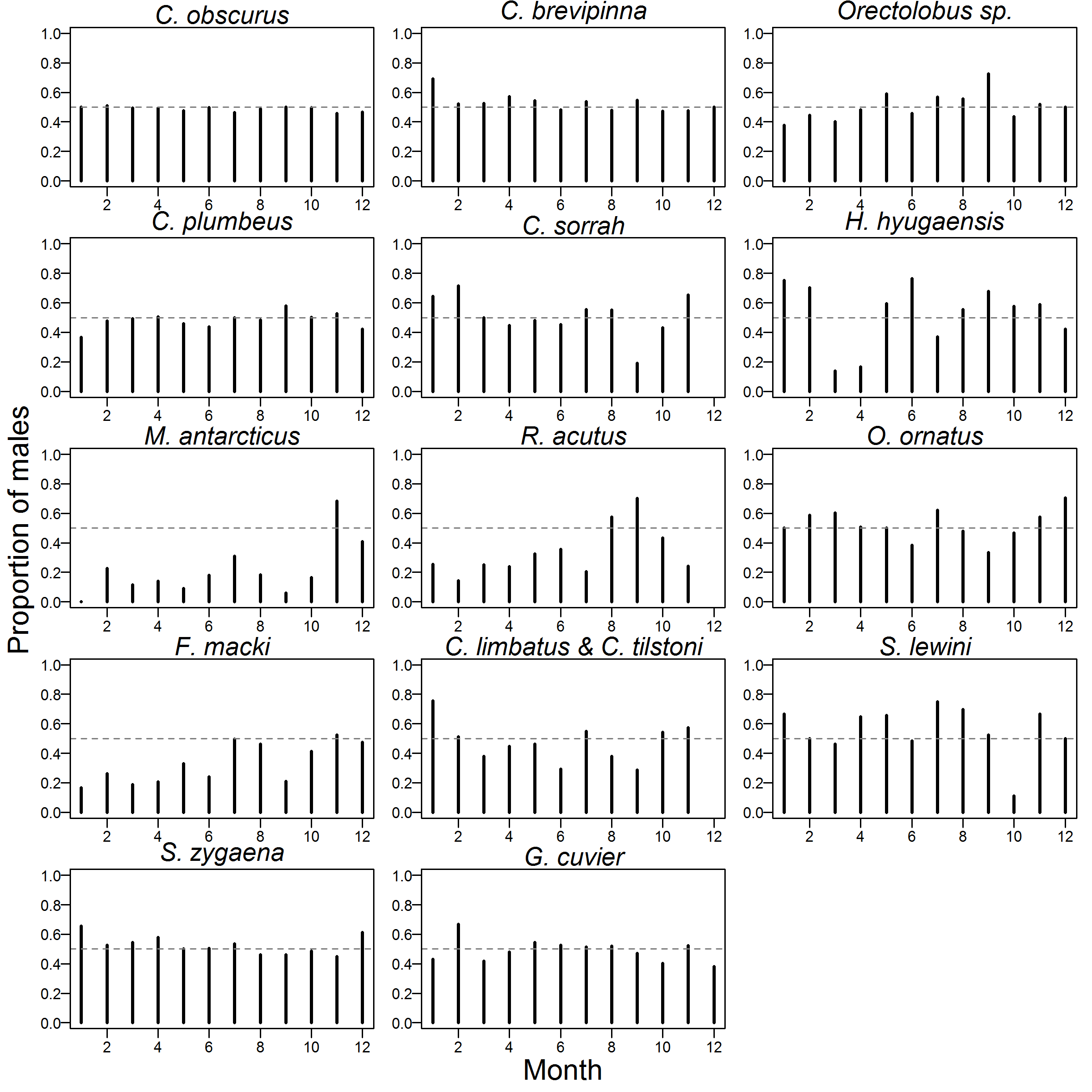

Supplement: Figure S6 [file rsos160306supp6.tiff]

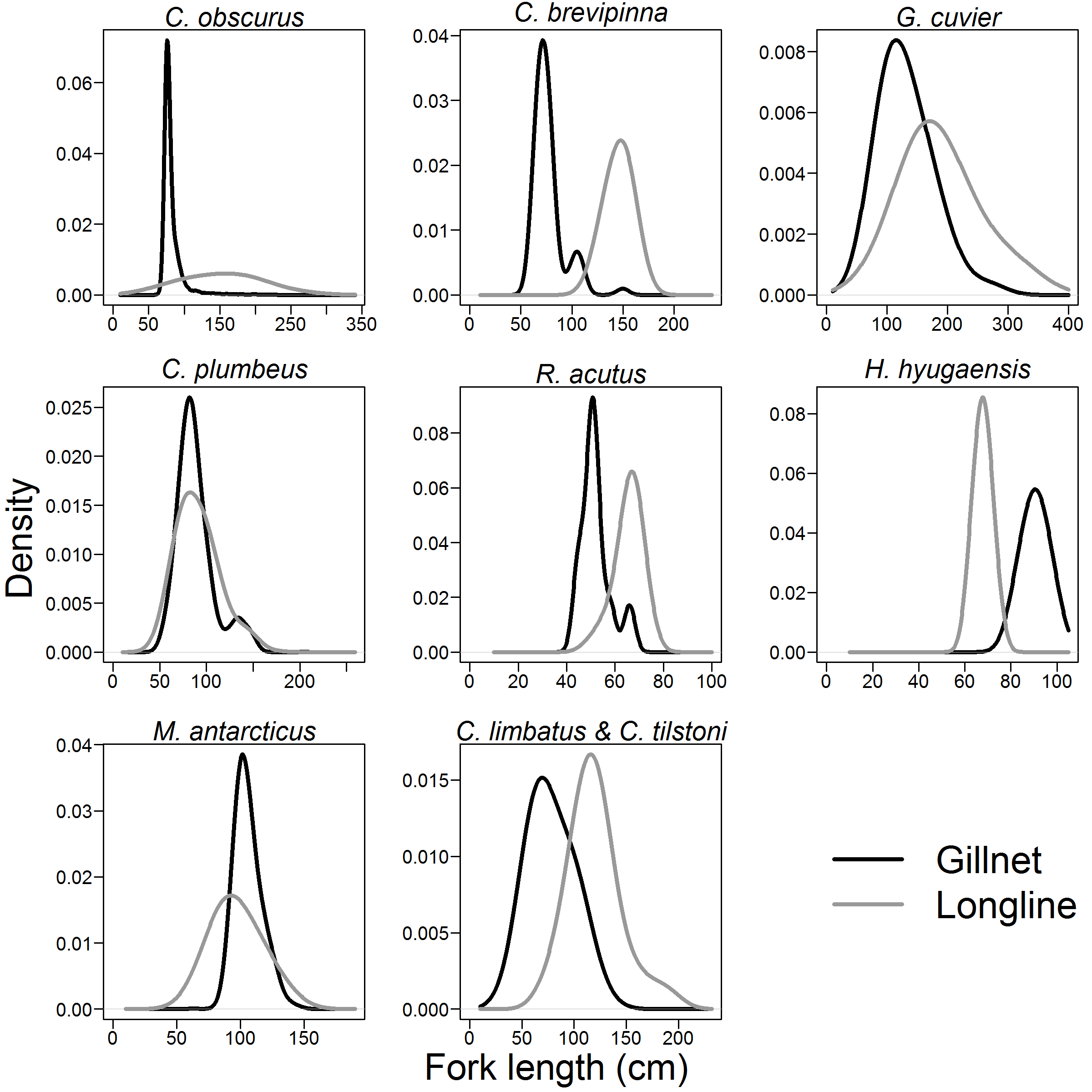

Supplement: Figure S7 [file rsos160306supp7.tiff]
